# *Cynara scolymus* Extract–Inulin Feed Additive Improves Intestinal Function and Growth in Weaned Piglets

**DOI:** 10.3390/ani16142250

**Published:** 2026-07-21

**Authors:** Guadalupe Martínez, Julieta María Decundo, Susana Nelly Diéguez, Denisa Soledad Pérez Gaudio, Carolina Paula Bianchi, Eugenio Colusi, Fabián Andrés Amanto, Alejandro Luis Soraci

**Affiliations:** 1 Laboratorio de Toxicología, Departamento de Fisiopatología, Facultad de Ciencias Veterinarias, Universidad Nacional del Centro de la Provincia de Buenos Aires (UNCPBA), Tandil B7000, Argentina; jdecundo@vet.unicen.edu.ar (J.M.D.); susadie@vet.unicen.edu.ar (S.N.D.); denisa@vet.unicen.edu.ar (D.S.P.G.); alejandro@vet.unicen.edu.ar (A.L.S.); 2 Centro de Investigación Veterinaria de Tandil (CIVETAN, UNCPBA-CICPBA-CONICET), Facultad de Ciencias Veterinarias, Tandil B7000, Argentina; cbianchi@vet.unicen.edu.ar; 3 Consejo Nacional de Investigaciones Científicas y Técnicas (CONICET), Buenos Aires C1425FQB, Argentina; 4 Comisión de Investigaciones Científicas de la Provincia de Buenos Aires (CICPBA), La Plata 1900, Argentina; 5 Laboratorio de Endocrinología, Departamento de Fisiopatología, Facultad de Ciencias Veterinarias, Universidad Nacional del Centro de la Provincia de Buenos Aires, Tandil B7000, Argentina; 6 Bedson S.A., La Lonja, Pilar, Buenos Aires 1629, Argentina; eugenio.colusi@bedson.com; 7 Área Producción Porcina, Departamento de Producción Animal, Facultad de Ciencias Veterinarias, Universidad Nacional del Centro de la Provincia de Buenos Aires, Tandil B7000, Argentina; famanto@vet.unicen.edu.ar

**Keywords:** weaned piglets, *Cynara scolymus*, inulin, intestinal functionality, growth performance

## Abstract

The early post-weaning period in piglets is characterized by physiological and environmental stress that can impair intestinal function and growth performance. Plant-derived feed additives have gained interest as a nutritional strategy to support gut integrity and productivity. Extracts from *Cynara scolymus* stimulate bile secretion and exhibit antioxidant properties, whereas inulin may promote beneficial microbial populations and intestinal functionality through prebiotic activity. This study evaluated the biological activity of a feed additive containing *Cynara scolymus* extract and inulin on intestinal functional indicators and productive performance of post-weaned piglets under intensive production conditions. Four hundred and eight piglets were randomly assigned to two groups: a control group (CON) that was fed a basal diet, and a treatment group (CSI) that was supplemented with the additive (5% *Cynara scolymus* extract and 14% inulin) at 300 g/ton of feed. Plasma cortisol and citrulline concentrations were measured during the first 15 days post-weaning. On day 16 post-weaning, intestinal parameters were evaluated, including gastrointestinal pH; enterobacteria and lactic acid bacteria counts; volatile fatty acids; bacterial adhesion to ileal mucus; disaccharidase activity; and small intestinal histomorphology. Growth performance was monitored during the 50-day nursery phase. Compared with CON piglets, CSI piglets exhibited higher plasma citrulline concentrations (72.72 ± 20.87 vs. 47.54 ± 17.05 µM; *p* < 0.001), indicating improved intestinal epithelial functionality. CSI supplementation also increased the villus height-to-crypt depth ratio (3.67 ± 0.75 vs. 2.68 ± 0.64; *p* = 0.001), suggesting enhanced intestinal epithelial integrity. Furthermore, CSI piglets showed higher average daily gain (0.53 ± 0.07 vs. 0.44 ± 0.09 kg/day; *p* = 0.003) and a better feed conversion ratio (1.27 ± 0.20 vs. 1.57 ± 0.57; *p* = 0.004). These results suggest that *Cynara scolymus* extract and inulin supplementation has beneficial biological effects on intestinal epithelial functionality and productive performance in post-weaning piglets under commercial conditions.

## Simple Summary

Weaning is one of the most stressful periods in pig production and can negatively affect intestinal health, growth, and animal welfare. For this reason, natural feed additives are increasingly being explored as alternatives to support gut health and reduce production losses. In this study, we evaluated the effects of a dietary supplement containing artichoke extract and inulin in weaned piglets raised under commercial farming conditions. Piglets receiving the supplement showed improved intestinal structure and better growth performance compared with untreated animals. These piglets also exhibited higher blood citrulline concentrations, a biomarker associated with intestinal epithelial functionality. No negative effects on digestive activity or intestinal microbial balance were observed. The results suggest that supplementation with a feed additive containing artichoke extract and inulin may contribute to improved intestinal functionality and productive performance in weaned piglets under intensive production conditions. These findings support the use of natural nutritional strategies to promote intestinal health in pig production systems.

## 1. Introduction

Nutritional strategies based on plant-derived additives have emerged as effective tools to alleviate intestinal disorders in stressed pigs. The abrupt dietary, social, and environmental transitions associated with early weaning induce significant stress compromising intestinal development, physiology, microbiota, and immunity in piglets [1,2,3]. Plant extracts have demonstrated the ability to ameliorate these intestinal impairments, thereby reducing the need for antimicrobials during the early post-weaning period [4,5]. Thus, during the past few years, the search for functional plant-derived additives has become a common practice in industrial pig farming. Among many different formulations, *Cynara scolymus* extract and inulin preparations have shown potential for enhancing young piglet performance in various studies [6,7,8,9].

*Cynara scolymus*, popularly known as globe artichoke, is cultivated worldwide and is used for its nutritional benefits and medicinal properties [10]. *Cynara scolymus* extract is obtained from its leaves and contains high levels of phenolic acids (combination of caffeic and quinic acid) and flavonoid compounds, particularly glycosides of luteolin [10,11,12]. Several studies in humans, birds, and other animal models demonstrated beneficial effects of *Cynara scolymus* phytochemical ingredients [13,14,15,16,17]. Nevertheless, studies including pigs are scarce. Previous studies conducted in pigs demonstrated that *Cynara scolymus* extract induces a choleretic and cholagogue effect in this species [18]. The increase in bile production observed in those studies provided a physiological basis for the beneficial effects of *Cynara scolymus* extract on gastrointestinal functionality in early-weaned piglets, as subsequently reported [19].

Inulin is a water-soluble, non-digestible oligosaccharide, mainly extracted from chicory (*Cichorium intybus*) and Jerusalem artichoke (*Helianthus tuberosus*) roots, and it is composed by a glucose molecule linked with a variable length chain of fructose molecules [20,21]. Dietary inulin is not hydrolyzed by gastrointestinal enzymes but is readily fermented by cecal and colonic bacteria. This prebiotic effect has shown to exert a beneficial impact on gastrointestinal health of pigs, both by modulating gut microbiota (promoting the growth of lactic acid bacteria while inhibiting enterobacteria) and by influencing the morphology and physiology of the intestinal epithelium (increasing villi height to crypt depth ratio and digestive enzymes activity) [22,23]. Furthermore, dietary inulin supplementation to piglets increase intestinal absorption of selenium, positively influence energy metabolism, and activate antioxidant enzymes [24]. These antioxidant properties are crucial to reduce the oxidative stress that occurs during the early post-weaning transition [25]. In some studies, the beneficial effect of dietary inulin on the gastrointestinal tract has been associated with increased growth performance [8,22,26].

As both *Cynara scolymus* extract and inulin have shown beneficial effects on the gut health and growth of young pigs by distinct mechanisms of action, we hypothesized that a formulation based on a combination of both plant extracts could provide enhanced efficacy to tackle stress-associated challenges during the early post-weaning transition. Accurate evaluation of the effects of dietary additives on animal health and performance requires experimental work conducted under real farm situations considering that managing tasks, environmental conditions, stocking density, and methods of administration may significantly modulate experimental outcomes. So, the aim of the present study was to investigate the impact of a feed additive containing *Cynara scolymus* extract and inulin on intestinal health and productive performance parameters in post-weaned piglets under an intensive production system.

## 2. Materials and Methods

### 2.1. Animals and Treatment

The present study was conducted on a commercial pig farm located in Buenos Aires Province, Argentina. All animal management and experimentation procedures were carried out in accordance with the Animal Welfare Guidelines of the University of the Center of Buenos Aires Province (UNCPBA). The experimental design of this study was evaluated and approved by the Animal Welfare Committee of the Faculty of Veterinary Sciences, UNCPBA (Resolution No. 087/02; exp 11/23), in compliance with EU directive 2010/63/EU.

Four hundred and eight clinically healthy, recently weaned piglets at 21 days of age from the same commercial genetic line and with a homogeneous weight of 5.51 ± 0.99 kg were selected. The study included castrated male and female piglets, which were similarly distributed between the experimental groups. The piglets were randomly assigned to one of the following groups: control piglets (n = 206; CON piglets) received a basal diet consisting of commercial feed (Biofarma S.A., Córdoba, Argentina), divided into 4 phases to cover the entire nursery phase (51 days). The digestible protein content decreased from 22% to 19%, and the metabolizable energy decreased from 14.2 to 14.0 MJ/kg from the beginning to the end of the nursing period (Table 1). Diets were formulated to meet or exceed nutrient requirements recommended for weaned pigs by the National Research Council in its publication entitled *Nutrient Requirements of Swine* [27]. The nutritional specifications presented in Table 1 correspond to calculated formulation values provided by the feed manufacturer (Biofarma S.A., Córdoba, Argentina). Detailed composition of the vitamin–mineral premix was not available because it is proprietary information of the manufacturer. *Cynara scolymus* extract–inulin-treated piglets (n = 202; CSI piglets) received a basal diet supplemented with a feed additive containing 5% *Cynara scolymus* extract and 14% inulin from chicory (*Cichorium intybus*) root (Bedson S.A.) at a dosage of 300 g/ton of feed. The experiment was conducted in two replicates separated by a 15-day interval. In each replicate, piglets were allocated to two separate pens within the same nursery room, with one pen assigned to the CON group (103 piglets per pen) and one pen assigned to the CSI group (101 piglets per pen). Environmental conditions were maintained under typical commercial nursery conditions (22 ± 5 °C; light–dark cycle 12:12 h; relative humidity 45–65%) and a stocking density of 0.3 m^2^/piglet. Twenty piglets from each group (10 from each replicate; same number of males and females) were randomly selected and identified using an ear tag for blood and intestinal sampling.

Routine management tasks were performed by farm personnel that was trained to monitor and register any abnormal behavior (vomiting, diarrhea, excitability, etc.) as well as mortality throughout the trial.

### 2.2. Sample Collection and Processing

#### 2.2.1. Plasma

Blood samples were collected in heparinized tubes by venipuncture of anterior vena cava at weaning (day 0) and 4, 8, 12, and 15 days later from all tagged piglets. Sampling began at 8:00 a.m. and took no longer than 30 min. On the weaning day (day 0), sampling took place 2 h after the piglets were separated from the sow. Blood samples were immediately centrifuged to obtain plasma, aliquoted and frozen at −20 °C until analyzed.

Plasmatic cortisol concentrations were measured using a commercial radioimmunoassay kit (IM 1841, Beckman Coulter, Inmunotech, Indianapolis, IN, USA). The sensitivity of the assay was 5 nM/L, and the intra-assay coefficient of variation was 5.14% for concentrations between 20 and 200 nM/L. All samples were measured in duplicate [28].

Plasma citrulline concentrations were quantified by HPLC-FLD following the method described by Wu and Meininger [29]. Briefly, 100 μL of plasma was mixed with methanol to precipitate proteins. After centrifugation, the clear supernatant was filtered through 0.22 µm nylon membranes and derivatized using OPA reagent. After 5.0 min the samples were injected into the HPLC system. For chromatographic separation, a C18 column (Phenomenex Luna, 250 × 4.6 mm; 5 µm) maintained at 30 °C was used. A gradient elution was performed using 50 mM sodium acetate buffer (pH 6.8) and a 2:1 mixture of methanol and acetonitrile as mobile phase. Excitation and emission wavelengths were 338 nm and 425 nm, respectively.

#### 2.2.2. Intestine

Six tagged piglets from each group (3 from each replicate) were randomly selected on day 16 post-weaning and were humanly euthanized using captive bolt stunning to the frontal region of the skull, inducing immediate unconsciousness, followed by jugular bleeding.

The pH was recorded using a digital pH meter (UP-25, Denver Instrument Company, Denver, CO, USA) in the caudal part of the stomach, 15 cm proximal to the ileocecal valve in the ileum, in the cecum, and 50 cm from the cecum in the colon.

Samples of cecal and ascending colon contents were collected in sterile tubes to evaluate enterobacteria (EBs) and lactic acid bacteria (LAB). Samples were refrigerated and processed in the laboratory within 4 h. One gram of intestinal content was diluted in 9 mL of peptone water and thoroughly homogenized. Serial dilutions were prepared, and aliquots were plated in duplicate. Lactic acid bacteria were cultured on de Man, Rogosa, and Sharpe (MRS) agar (Britania S.A., Ciudad Autónoma de Buenos Aires, Argentina), while EBs were cultured on MacConkey agar (Britania S.A., Ciudad Autónoma de Buenos Aires, Argentina) [30,31,32]. Plates were incubated at 37 °C, and colony-forming units (CFUs) were counted after 24 h. Results were expressed as log_10_ CFU/g of intestinal content. The EB:LAB ratio was calculated as an indicator of intestinal microbial balance.

Volatile fatty acids (VFAs) were determined in cecal contents. Samples were collected in sterile tubes and immediately diluted in phosphoric acid (4:1, *v*/*v*), then stored at −70 °C until analysis. Samples were weighed and diluted with methanol, centrifuged, and filtered. Volatile fatty acid concentrations were determined by gas chromatography, following the method described by Jouany [33]. A Shimadzu GCe17A gas chromatograph (Kyoto, Japan) equipped with an FID detector and a 30 m Innowax 19091N-133 capillary column (Agilent, Santa Clara, CA, USA) was used for VFAs separation and quantification. Calibration curves were prepared using a 10 mmol/L Supelco standard mixture (C2–C10) and 2-ethylbutyric acid (Fluka, Charlotte, NC, USA) as the internal standard.

Ten cm segments were harvested from the mid-jejunum (3 m distal from pylorus) and ileum (15 cm proximal to ileocecal valve). Intestinal segments were rinsed with sterile saline solution (NaCl 0.9%) and fixed in buffered formalin. Paraffin embedded tissues were sliced and stained with hematoxylin and eosin for histological analysis. Tissue sections were examined under a light microscope (Olympus BX40, Tokyo, Japan), and 50 villi height (Vh) and associated crypt depth (Cd) per histological section were measured using the Image Analysis Software ToupTek-View version 4.12. Villus height over crypt depth ratio (Vh:Cd) was used as indicator of intestinal functionality.

The quality of the mucus was evaluated based on its ability to bind to pathogenic *Escherichia coli*, using the method described by Bai et al. [34] with slight modifications. Ileal mucus was obtained by gentle mucosal scraping, and glycoproteins were extracted and sterilized by filtration. The mucus extract was then incubated with *E. coli* O157:H7 (10^3^ CFU/mL) at 37 °C with agitation. After incubation, adherent and non-adherent bacteria were separated by differential centrifugation and plated onto sorbitol MacConkey agar (Britania S.A., Ciudad Autónoma de Buenos Aires, Argentina) for colony enumeration. The results were expressed as a percentage of the bacteria that adhered to the intestinal mucus.

Brush border disaccharidase activities were measured in the jejunum and ileum. Intestinal samples were collected from the proximal jejunum and ileum. Each segment was rinsed with physiological saline to remove luminal contents and opened along the mesenteric border, and the mucosa was scraped. Mucosal scrapings were stored at −70 °C until processing. For homogenate preparation, 1 g of mucosal tissue was diluted in physiological saline and homogenized sequentially using an Ultra-Turrax and a Potter-Elvehjem homogenizer. The homogenates were then centrifuged at 4825× *g* for 10 min at 4 °C, and the supernatant was aliquoted and stored at −70 °C until enzymatic analysis. Protein concentration in the homogenates was determined using the Bradford assay [35], with bovine serum albumin as a standard. Disaccharidase activities (lactase, sucrase, and maltase) was quantified according to the Dahlqvist method [36], based on the amount of glucose released. Diluted homogenates were incubated with 56 mM/L lactose, sucrose, or maltose for 1 h at 37 °C. After incubation, a glucose reagent mixture (glucose oxidase, peroxidase, O-dianisidine, Triton X-100, and Tris buffer) was added. Absorbance was measured at 450 nm using a spectrophotometer (Dupont, Sorvall Instruments, Wilmington, DE, USA). Enzyme activity was expressed as units (U)/mg of protein, where one U is defined as the amount of enzyme required to hydrolyze 1 nmol of disaccharide per minute under assay conditions.

### 2.3. Piglets Performance

Piglets were individually weighted at weaning and 20 and 50 days post-weaning. Average daily gain during the nursery phase (ADG_0–50_) was calculated for the identified piglets. Feed intake was recorded daily at the pen level under commercial farm conditions. Average daily feed intake (ADFI_0–50_) was calculated for each experimental group, and the feed conversion ratio (FCR_0–50_) was estimated from pen feed intake and individual body weight gain.

### 2.4. Statistical Analysis

R Studio^®^ software version 4.5.0 was used for statistical analysis. Normality and homogeneity of variance were assessed using the Shapiro–Wilk and Bartlett tests, respectively.

Cortisol and citrulline concentrations were analyzed using linear mixed models, including treatment, day, and their interaction as fixed effects, and piglet as a random effect to account for repeated measurements within the same animal. The same linear mixed model was applied to analyze pH, disaccharidase activities, and histomorphological parameters, including treatment, intestinal zone, and their interaction as fixed effects, and piglet as a random effect. When significant effects were detected (*p* < 0.05), post hoc multiple comparisons were performed using Tukey’s or Dunn’s tests, as appropriate.

To assess cortisol and citrulline production during the early post-weaning period, the area under the concentration–time curve (AUC) was calculated for cortisol (nmol·day/L; AUCcor) and citrulline (µmol·day/L; AUCcit) using non-compartmental pharmacokinetic analysis software (PK Solutions version 2.0; Summit Research Services Co., Montrose, CO, USA, Farrier, 1997).

AUCcor, AUCcit, bacterial counts, EB:LAB ratio, VFA concentrations, percentage of bacteria adhering to the intestinal mucus, body weight, ADG_0–50_, and FCR_0–50_ were analyzed using Student’s *t*-test or the Mann–Whitney U test, as appropriate, to evaluate differences between groups.

## 3. Results

Plasma cortisol concentrations were not affected by treatment or by interactions between treatment and sampling day (*p* > 0.05). An effect of sampling day was observed (*p* < 0.01): plasma cortisol concentrations peaked at weaning, remained as high 4 days post-weaning, and declined towards 8 and 12 days post-weaning. Both groups showed similar AUCcor (Table 2).

Significant effects of sampling day, treatment, and their interaction on plasma citrulline concentrations were observed (*p* < 0.001). Both CON and CSI piglets exhibited similar plasma citrulline concentrations at weaning. Plasma citrulline concentrations decreased sharply from weaning to 4 and 8 days later, rebounded at 12 days post-weaning, and reached weaning levels by 15 days post-weaning. Across all sampling points (days 4, 8, 12, and 15) CSI piglets consistently displayed higher citrulline levels than CON piglets. CSI piglets showed significantly higher AUCcit than CON piglets (Table 3).

Gastrointestinal pH was not affected by treatment (*p*: 0.938) or by interactions between treatment and gastrointestinal zone (*p*: 0.901). However, a zone effect was observed (*p* < 0.001), with the lowest pH recorded in the stomach and the highest in the ileum. No statistically significant differences were found between the cecum and the colon. Mean pH values (±SD) were 3.27 ± 1.49 in the stomach, 7.01 ± 0.56 in the ileum, 6.50 ± 0.73 in the cecum, and 6.11 ± 0.47 in the colon.

Enterobacteria and lactic acid bacteria counts, as well as the EB:LAB ratio, were not significantly affected by treatment (*p* > 0.05). Similarly, no statistically significant differences were observed in the concentrations of individual VFAs, including acetic, propionic, butyric, and valeric acids, or in total VFA concentrations between treatments (*p* > 0.05; Table 4).

Villi height was not affected by the treatment or by interactions between treatment and intestinal zone (*p* > 0.05); however, Vh in the jejunum were higher than Vh in the ileum, showing a zone effect (*p*: 0.006).

Crypt depth was not affected by the intestinal zone or by interactions between the treatment and intestinal zone (*p* > 0.05); however, the treatment resulted in lower Cd in CSI piglets compared with CON piglets (*p* < 0.001).

No interactions were observed between intestinal zone and treatment on Vh:Cd (*p*: 0.830). However, effects of the intestinal zone (the jejunum showed higher Vh:Cd than ileum; *p*: 0.002) and of the treatment (CSI piglets showed higher Vh:Cd than CON piglets; *p*: 0.001) were observed (Table 5).

Adherence of pathogenic *E. coli* to glycoproteins to intestinal mucus was not affected by treatment (*p*: 0.056). Mean adherence values were 43.67 ± 12.97% in CON piglets and 62.17 ± 16.48% in CSI piglets.

Lactase, sucrase, and maltase activities were not affected by treatment or by the interaction between treatment and intestinal zone (*p* > 0.05; Table 6).

Behavioral patterns and mortality were similar in both groups, as typically observed in the farm, indicating that the treatment did not cause any observable adverse effects on the general health and welfare of the animals.

Control and CSI piglets exhibited similar weight at weaning and 20 days later (*p* > 0.05). Nevertheless, CSI piglets showed higher weight (*p*: 0.001), higher ADG_0–50_ (*p*: 0.003) and lower FCR_0–50_ (*p*: 0.004) than CON piglets (Table 7) at the end of nursery phase.

## 4. Discussion

Dietary plant-derived extracts have become a valuable tool to cushion the detrimental impact of stress associated with early weaning in intensive pig production systems. To fully understand the efficacy of dietary additives on animal health and performance, it is essential to assess their effects under real farm conditions. In the present work, we have demonstrated that the strategic incorporation of a novel combination of *Cynara scolymus* extract and inulin from chicory root to the diet enhances the intestinal health and growth performance of nursery piglets on an intensive pig production farm.

Even if responses to stress are idiosyncratic, the increase of plasma cortisol is a common feature during stressful situations. Thus, plasma cortisol concentrations can be used as indicator of stress and animal welfare [37]. In our study, the piglets exhibited peak cortisol levels on weaning day, likely due to stress caused by the recent separation from the sow and restraint for weighing. Such practices have proven to stress the animals, resulting in a cortisol surge within 2 to 3 h [38,39,40]. The persistent high plasma cortisol concentrations observed four days post-weaning reflect the transition through the acute phase of weaning stress, which can last for about a week [37,41]. During this period, the adaptation to a new environment, routine, feed, and social group, characterized by aggressive interactions to establish social hierarchy [42,43], generate high stress for the young piglets [37,44]. Thereafter, plasma cortisol levels declined from day 8 post-weaning onward, as the piglets adapted to their new conditions. Furthermore, dietary CSI did not induce additional stress, as plasma cortisol levels and overall cortisol production (AUCcor) remained within the expected range for nursery piglets during the first 12 days post-weaning [45,46] in both groups.

Plasma citrulline concentrations were measured as a biomarker of intestinal functionality because circulating citrulline reflects the functional mass and metabolic activity of enterocytes [47,48,49]. In both groups, citrullinemia decreased sharply from weaning to indicating a reduction of intestinal function 4 and 8 days later, during the acute phase of weaning stress. Our results align with those obtained by Soraci et al. [47], who reported that reduced feed intake and potential anorexia exacerbate the negative effects of inflammation on intestinal function by limiting amino acids availability for citrulline synthesis [50,51]. It is well documented that certain natural feed additives, like probiotics, prebiotics, essential oils, or organic acids, can compensate, to some extent, for the intestinal damage caused by early weaning through mechanisms that produce anti-inflammatory, antioxidant, and trophic effects on the intestinal mucosa and favor the development of beneficial bacteria populations [52,53,54]. A prior investigation conducted by our research group revealed that oregano essential oils attenuated the decline in citrullinemia during the acute phase of post-weaning stress in piglets. This beneficial effect was supported by improvements in villus–crypt architecture and mucus quality, elevated intestinal disaccharidase activities, increased short chain fatty acids production, and enhanced global productive performance [55]. In the current study, citrullinemia levels were similar between CSI and CON piglets at weaning, but thereafter (days 4, 8, 12, and 15), CSI piglets exhibited consistently higher citrullinemia and overall citrulline production (higher AUC_0–15_) than CON piglets, indicating that the dietary supplementation of CSI positively influenced intestinal function. Several mechanisms may contribute to the higher citrullinemia observed in CSI piglets. Among them, the increased bile acid availability associated with *Cynara scolymus* extract supplementation could play a relevant role [18]. Bile acids exert a direct trophic action on the intestinal epithelium. Bile acids act on G protein-coupled bile receptor (TGR), found in enteroendocrine cells, to stimulate the secretion of glucagon-like peptide 1 (GLP 1) and glucagon-like peptide 2 (GLP 2) [56,57,58,59]. Glucagon-like peptide 1 is the major hormone involved in glucose homeostasis, and GLP 2 is a key trophic hormone in intestinal adaptation and growth in response to food ingestion. Moreover, GLP 2 enhances digesta transit time, blood flow, intestinal barrier, and Paneth cell-mediated immune protection, which leads to lower inflammation and intestinal damage [60,61,62]. At the same time, bile acids activate the enterocytes’ farnesoid nuclear receptor X (FXR), promoting anti-inflammatory and antioxidant effects by inhibition of the NF-κB pathway [63,64]. On the other hand, inulin from chicory root regulates energy metabolism and activates enzymatic systems that maintain redox homeostasis in the liver of growing pigs [25,65]. In this way, a milder decline of citrullinemia during the acute phase of weaning stress and a sooner recovery to pre-weaning values would represent greater general health benefits in CSI piglets than in CON piglets, which by 15 days post-weaning had still not recovered plasma citrulline pre-weaning levels.

In addition, both inulin and bile acids have been reported to modulate gut microbial populations by promoting beneficial microorganisms and inhibiting detrimental ones [66,67]. In contrast to previous reports showing that inulin supplementation can modulate gut microbiota and increase VFA production in weaned pigs [20,23], in the present study there were no significant differences in enterobacteria, lactic acid bacteria counts, EB:LAB ratio, or cecal VFA concentrations. However, it should also be noted that the microbial analysis was limited to culture-based enumeration of EBs and LAB. Therefore, potential changes in the overall microbial community structure, diversity, and abundance of specific taxa may have gone undetected as no molecular characterization of the microbiota (e.g., 16S rRNA gene sequencing, alpha and beta diversity analyses, or taxonomic profiling) was performed.

In addition to microbial counts, VFAs provide relevant functional information regarding intestinal microbial activity. Volatile fatty acids are key metabolites in intestinal physiology, serving as energy substrates for enterocytes and contributing to epithelial integrity, immune modulation, and barrier function [3,68]. 

On the other hand, the absence of detrimental shifts in fermentation patterns indicates that CSI supplementation did not compromise luminal microbial homeostasis under commercial conditions. Therefore, the improvements in citrullinemia and intestinal morphology observed in CSI piglets are more likely attributable to the trophic and metabolic effects associated with enhanced bile acid signaling and epithelial functionality rather than to major modifications of luminal fermentation.

The better functioning intestinal epithelium of CSI piglets compared with CON piglets, as shown by citrullinemia levels, was also reflected in the higher Vh:Cd observed in the former group. This effect was due to greater villus cells turnover (deeper crypts) in CON piglets. At the time of sampling (16 days post-weaning), CON piglets exhibited a sustained demand for cell renewal to maintain Vh, whereas CSI piglets showed a superior ability to preserve Vh with shorter Cd. It is likely that in our study the mechanisms triggered by inulin and bile acids (as a result of the intake of *Cynara scolymus* extract) act together to enhance intestinal health in CSI piglets.

Although no treatment effect was observed on bacterial adhesion to ileal mucus, this parameter provides relevant information on mucosal barrier function. Goblet cells continuously secrete mucins that form the outer mucus layer, which can competitively bind bacterial adhesins and prevent direct interaction of microorganisms with epithelial cell receptors [69]. Bacteria retained within the mucus layer are physically separated from the epithelium, thereby limiting epithelial colonization and pathogen proliferation. In the present study, the absence of differences between treatments suggests that CSI supplementation did not disrupt mucus–bacteria interactions and preserved mucosal barrier integrity.

Disaccharidase activities exhibited considerable inter-animal variability in the present study, which should be taken into account when interpreting the results. Such variation is likely multifactorial and may be related to differences among animals in birth weight, colostrum intake, intestinal maturation, and gut microbiota composition [70]. The lack of treatment effect on disaccharidase activities suggests that the improvement in intestinal health observed in CSI piglets was not associated with changes in brush border digestive capacity at the evaluated time point. 

Ultimately, CSI piglets exhibited enhanced nursery phase performance, characterized by increased weight gain and average daily gain alongside improved feed efficiency compared with CON piglets. The association between nursery phase performance and overall productive performance is well established [71,72].

The experimental design did not include groups supplemented separately with *Cynara scolymus* extract or inulin. Therefore, the relative contribution of each component to the observed effects cannot be determined. Consequently, the results should be interpreted as the response to the combined feed additive evaluated under commercial production conditions rather than as evidence of the individual effects of either component. Future studies including separate treatment groups would help clarify the specific mechanisms associated with each ingredient.

Taken together, dietary supplementation with CSI improved intestinal structural integrity, enhanced metabolic markers of epithelial functionality, and resulted in superior growth performance under commercial production conditions. These findings highlight the potential of this natural feed additive as a practical nutritional strategy to enhance intestinal health and productivity in intensive pig production systems. From a production perspective, improvements in average daily gain and feed efficiency may translate into tangible economic benefits for commercial farms. Moreover, the use of plant-derived functional additives represents a promising approach to enhance intestinal health during the critical post-weaning period and may contribute to reducing reliance on prophylactic antibiotic use in swine production.

## 5. Conclusions

Supplementation with a feed additive based on *Cynara scolymus* extract and inulin improved intestinal epithelial functionality and productive performance in post-weaning piglets raised under commercial conditions. The inclusion of this natural additive was associated with higher plasma citrulline concentrations, improved villus height-to-crypt depth ratios, greater average daily gain, and better feed efficiency without negatively affecting intestinal microbial indicators, fermentation activity, or digestive enzyme activity. These findings support the use of natural nutritional strategies to promote intestinal health and productivity during the critical post-weaning period and highlight the potential of *Cynara scolymus* extract and inulin as alternatives to support sustainable pig production systems.

## Figures and Tables

**Table 1:** Composition of basal diet (as fed, % of the diet).

Ingredient	Content
Corn	66.670
Soybean pellet	23.330
Soybean expeller	6.670
Total lysine	1.620
Total methionine	0.437
Total tryptophan	0.275
Total threonine	1.085
Total calcium	0.888
Total phosphorus	0.710
Lactose	12.000
Sodium	0.280
Chloride	0.250
Vitamin and mineral	3.330
Analyzed composition	
Minimum protein content	20.00
Maximum humidity	12.00
Maximum mineral content	38.00
Ether extract	2.50
Maximum crude fiber	2.00
Minimum calcium content	8.00
Minimum total phosphorous content	4.00
Average available phosphorous	3.80

**Table 2:** Effect of dietary treatments on plasma cortisol concentrations (ng/mL).

Days	CON	CSI	Day Mean
0	76.62 ± 17.78	65.58 ± 23.95	70.90 ± 21.55 ^A^
4	64.21 ± 21.21	58.64 ± 12.59	61.32 ± 17.17 ^AB^
8	46.13 ± 23.80	55.83 ± 17.30	51.16 ± 20.87 ^B^
12	50.14 ± 15.52	49.25 ± 19.94	49.68 ± 17.61 ^B^
Treatment mean	59.27 ± 22.77	57.33 ± 19.31	
AUCcor	1919.54 ± 357.80	1899.14 ± 397.06	

^A,B^ within column: mean values with different superscript letter differ significantly (*p* < 0.05). Treatment *p* = 0.654. Day *p* < 0.01. Treatment × Day *p* = 0.189. CON: control piglets; CSI: *Cynara scolymus* extract–inulin piglets; AUCcor: area under the cortisol concentration–time curve.

**Table 3:** Effect of dietary treatments on plasma citrulline concentrations (µM).

Days	CON	CSI	Day Mean
0	66.49 ± 9.35 ^A^	76.81 ± 16.57 ^A^	71.65 ± 14.22 ^A^
4	34.86 ± 10.97 ^aB^	55.47 ± 14.49 ^bB^	45.16 ± 16.18 ^B^
8	35.52 ± 9.53 ^aB^	65.21 ± 19.35 ^bB^	50.75 ± 21.25 ^B^
12	47.68 ± 16.16 ^aC^	76.85 ± 17.11 ^bA^	61.89 ± 21.73 ^C^
15	52.80 ± 17.60 ^aC^	89.44 ± 20.22 ^bC^	71.59 ± 26.14 ^A^
Treatment mean	47.54 ± 17.05 ^a^	72.72 ± 20.87 ^b^	
AUCcit	657.95 ± 139.43 ^a^	1041.54 ± 229.08 ^b^	

^a,b^ within a row and ^A–C^ within column: mean values with different superscript letter differ significantly (*p* < 0.05). Treatment *p* < 0.001. Day *p* < 0.001. Treatment × Day *p* < 0.001. CON: control piglets; CSI: *Cynara scolymus* extract–inulin piglets; AUCcit: area under the citrulline concentration–time curve.

**Table 4:** Effect of dietary treatments on intestinal bacterial counts (log10 CFUs) and volatile fatty acid concentrations (mM).

Variable	CON	CSI	*p*-Value
Bacteria			
Enterobacteria	8.23 ± 0.34	8.50 ± 0.35	0.067
Lactic acid bacteria	9.30 ± 0.59	9.75 ± 0.51	0.060
EB:LAB ratio	0.88 ± 0.04	0.87 ± 0.05	0.470
VFAs			
Acetate	41.82 ± 27.16	52.89 ± 11.04	0.377
Propionate	20.28 ± 13.46	23.45 ± 4.14	0.593
Butyrate	7.61 ± 4.93	7.95 ± 1.66	0.876
Valerate	2.24 ± 0.98	2.34 ± 0.39	0.818
Total VFAs	72.62 ± 46.17	89.49 ± 15.06	0.823

CON: control piglets; CSI: *Cynara scolymus* extract–inulin piglets; EB: enterobacteria; LAB: lactic acid bacteria; VFA: volatile fatty acids.

**Table 5:** Effect of dietary treatments on villus height (Vh), crypt depth (Cd), and villus height over crypt depth ratio (Vh:Cd).

Variable	Zone	CON	CSI	Zone Mean	*p*-Value		
					Treatment	Zone	Treatment × Zone
Vh					0.290	0.006	0.777
	Jejunum	328.53 ± 58.49	355.24 ± 52.54	341.89 ± 54.8 ^A^			
	Ileum	287.36 ± 41.72	304.52 ± 25.21	295.94 ± 34.07 ^B^			
	Treatment mean	307.94 ± 53.00	329.88 ± 47.38				
Cd					<0.001	0.293	0.430
	Jejunum	112.52 ± 15.26	90.47 ± 11.52	101.49 ± 17.28			
	Ileum	121.13 ± 12.97	91.70 ± 9.65	106.41 ± 18.84			
	Treatment mean	116.82 ± 14.23 ^a^	91.09 ± 10.15 ^b^				
Vh:Cd					0.001	0.002	0.830
	Jejunum	2.97 ± 0.69	4.00 ± 0.95	3.48 ± 0.96 ^A^			
	Ileum	2.40 ± 0.49	3.33 ± 0.22	2.87 ± 0.61 ^B^			
	Treatment mean	2.68 ± 0.64 ^a^	3.67 ± 0.75 ^b^				

^a,b^ within row and ^A,B^ within column: mean values with different superscript letter differ significantly (*p* < 0.05). CON: control piglets; CSI: *Cynara scolymus* extract–inulin piglets.

**Table 6:** Effect of dietary treatments on disaccharidase activities (U/mg protein).

Variable	Zone	CON	CSI	Zone Mean	*p*-Value		
					Treatment	Zone	Treatment × Zone
Lactase					0.168	<0.001	0.078
	Proximal jejunum	1837.39 ± 1255.77	1013.98 ± 272.92	1425.69 ± 967.25 ^A^			
	Ileum	128.24 ± 82.20	180.40 ± 151.79	154.32 ± 119.52 ^B^			
	Treatment mean	1001.90 ± 1289.73	597.19 ± 483.57				
Sucrase					0.176	<0.001	0.200
	Proximal jejunum	1408.40 ± 355.54	1451.52 ± 302.80	1429.96 ± 315.66 ^A^			
	Ileum	2597.37 ± 445.11	3641.53 ± 1831.01	3119.45 ± 1382.50 ^B^			
	Treatment mean	2073.76 ± 721.16	2546.52 ± 1695.17				
Maltase					0.074	0.936	0.559
	Proximal jejunum	2064.05 ± 717.32	2573.47 ± 604.58	2318.76 ± 686.15			
	Ileum	1854.11 ± 749.26	2732.88 ± 1255.69	2293.49 ± 1087.42			
	Treatment mean	2023.60 ± 704.44	2653.17 ± 943.28				

^A,B^ within column: mean values with different superscript letter differ significantly (*p* < 0.05). CON: control piglets; CSI: *Cynara scolymus* extract–inulin piglets.

**Table 7:** Effect of dietary treatments on the main zootechnical parameters.

Variable	CON	CSI	*p*-Value
Weaning weight (kg)	5.49 ± 0.84	5.53 ± 1.01	0.493
Weight at 20 days post-weaning (kg)	10.40 ± 1.95	10.51 ± 2.49	0.896
Weight at 50 days post-weaning (kg)	26.20 ± 5.02 ^a^	28.10 ± 5.80 ^b^	0.001
ADG_0–50_ (kg/d/pig)	0.44 ± 0.09 ^a^	0.53 ± 0.07 ^b^	0.003
ADF_I0–50_ (kg feed)	31.80 ± 0.78	32.60 ± 0.85	
FCR_0–50_ (kg feed/kg B.W. gain)	1.57 ± 0.57 ^a^	1.27 ± 0.20 ^b^	0.004

^a,b^ within row: mean values with different superscript letter differ significantly (*p* < 0.05). CON: control piglets; CSI: *Cynara scolymus* extract–inulin piglets; ADG: average daily gain; ADFI: average daily feed intake; FCR: feed conversion ratio.

## Data Availability

The original contributions presented in this study are included in the article. Further inquiries can be directed to the corresponding author.
